# The value of 2D speckle-tracking strain echocardiography in evaluating the relationship between carotid elasticity and left ventricular systolic function in patients with diabetic nephropathy

**DOI:** 10.1186/s13244-020-00897-0

**Published:** 2020-08-17

**Authors:** Xiuyun Li, Hongju Kou, Yanyan Dong, Chao Zheng, Pengfei Wang, Maosheng Xu, Chunpeng Zou, Liang Wang

**Affiliations:** 1 Department of Ultrasonic Diagnosis, The Second Affiliated Hospital and Yuying Children’ s Hospital of Wenzhou Medical University, No.109 West Xueyuan Road, Wenzhou, 325027 Zhejiang China; 2 Department of Endocrinology, The Second Affiliated Hospital of Zhejiang University School of Medicine, Hangzhou, 310000 China

**Keywords:** Diabetic nephropathy, Common carotid artery, Ultrasound, Longitudinal strain, 2D speckle-tracking strain echocardiography

## Abstract

**Objective:**

To investigate the relationship between the elasticity of the carotid artery and the LV (left ventricle) systolic function in patients with diabetic nephropathy (DN) by using two-dimensional speckle-tracking strain echocardiography (2D-STE).

**Methods:**

DN patients (*n* = 108) and control subjects (*n* = 112), all of whom underwent echocardiography and carotid ultrasound. Analysis of LV GLS (global longitudinal strain) from the apical two-chamber (2C), three-chamber (3C), and four-chamber (4C) views. Meanwhile, the circumferential strain (CS) of the carotid artery was obtained from the view of the short-axis right common carotid artery. The differences between the two groups were compared, and a correlation analysis between CS and GLS was performed.

**Results:**

The 4CGLS, 2CGLS, 3CGLS, and CS of the DN group were significantly lower at significant levels in contrast to the control group (*p* < 0.05). There was a significantly positive correlation of CS with 4CGLS, 2CGLS, and 3CGLS in all subjects (*r* = 0.809, *p* = 0.000; *r* = 0.830, *p* = 0.000; *r* = 0.830, *p* = 0.000, respectively).

**Conclusion:**

2D-STE is a relatively new technique for assessing the mechanical characteristics of the carotid artery in patients with DN. Reduced values of CS correlate with reduced LV systolic function as evaluated by strain measurements, which can predict the risk of systolic dysfunction of LV.

## Key points

• Increased arterial stiffness measured by 2D-STE is indicative of a potential link between vascular changes and LV systolic function.

• Reduced CS values correlate with reduced LV systolic function evaluated by strain measurements.

• 2D-STE can detect the relationship between CS and GLS in the early stage.

## Introduction

Diabetes mellitus (DM) is a serious threat to human health and life. It can lead to a variety of complications [1–3]. Among all the complications of DM, cardiovascular complications are important causes of disability and death [4], and diabetic nephropathy (DN) is the major cause of end-stage renal failure. Renal disease is also an important cause of cardiovascular disease (CVD), including sudden cardiac death and stroke [5, 6]. Thus, DN patients have a high risk of CVD, which is identified as the leading reason of death in these patients.

It was reported that stiffness in arteries was a major and robust independent predictor of CVD [7, 8]. Arterial stiffness was recommended as a proof of damage to the target organs in the European guidelines for the hypertension diagnosis and treatment [9]. There are several methods of evaluating arterial stiffness, such as vascular catheterization, ultrasound, magnetic resonance imaging [10], and arterial tonometry [11]. 2D-STE (two-dimensional speckle-tracking strain echocardiography) is a useful technology that has been developed in recent years [12, 13]. It is an accurate, angle-independent, and noninvasive method for evaluating cardiac function [14]. It can be used to obtain myocardial deformation by tracking intramyocardial speckles, accordingly calculating the strain (S) and strain rate (Sr) of the myocardium [15]. This strain-based imaging technique has been shown to have clinical utility in a variety of settings [16]. It can predict severe coronary artery disease (CAD) in women with normal LV function [17]. It can be used to identify the ischemic etiology of LV systolic dysfunction [18]. The study by Atici et al. showed that GLS (global longitudinal strain) evaluated through 2D-STE is a potential method for predicting CAD in patients with non-ST-segment elevation myocardial infarction [19]. 2D-STE can assess the impairment of left atrial phasic function in patients with heart failure with mid-range ejection fraction (EF) [20]. At present, this technique is also used to evaluate the elasticity of the carotid artery, and the obtained strain has a good correlation with carotid elasticity [21]. The aortic circumferential strain and the rate of strain estimated by 2D strain imaging enable accurate and simple evaluation of the stiffness of the aorta [22]. However, the relationship between the elasticity of the carotid artery and the systolic function of the LV remains unclear. The study aimed to examine the association between carotid elasticity and LV systolic function in DN patients using 2D-STE.

## Materials and methods

### Patients

Between May 2017 and November 2019, 139 patients newly diagnosed with DN participated in this study. DN was diagnosed according to the Tervaert criteria [23]. The exclusion criteria were as follows: ①patients with congenital vascular disease and ② patients with vascular diseases secondary to hypertension, hyperlipidemia, cardiac dysfunction, or endocrine diseases. The final study consisted of 108 patients (62 women, 46 men, mean age 50.16 ± 12.30 years). Meanwhile, 112 healthy individuals served as a control group (67 women, 45 men, mean age 47.41 ± 9.72 years). All subjects did not suffer from any CVD or associated risk factors that were known. Coffee and alcohol were not administered within 24 h before examination.

All of the above candidates received detailed clinical evaluation and biochemical tests. Detailed clinical evaluation items included medical history, height, weight, BMI (body mass index), blood pressure, and cardiovascular examination. Biochemical test items included FBG (fasting blood glucose), HbA1c (hemoglobin A1c), TG (triglycerides), TC (total cholesterol), HDL-C (high-density lipoprotein cholesterol), and LDL-C (low-density lipoprotein cholesterol).

### Carotid ultrasound

Carotid ultrasound was performed with a GE Vivid E9 (GE Healthcare) ultrasound system, equipped with a 10-L probe (frequency ranged 7.5~10 MHz) and M4S probe (frequency ranged 1.5~5.0 MHz). Digitized images of the right common carotid artery (CCA) were obtained by 10 L probe. Carotid IMT (intima-media thickness) was measured in right CCA at end diastole, 1.0 cm proximal to the carotid bulb [24, 25]. Plaque formation was defined as IMT ≥ 1.2 mm [26]. Patients with presented plaque were excluded from this study. Color Doppler ultrasonography was used to detect PSV (peak flow velocity), EDV (end-diastolic flow velocity), PI (pulsatility index), and RI (resistance index) of the carotid artery. Then, an inflatable balloon containing 100 ml saline was used to increase the surface contact and improve the acoustic window, and the equipment was switched to the M4S probe. Dynamic images of the short axis of the carotid artery were obtained and stored for up to three consecutive cardiac cycles.

### Echocardiography

Echocardiography was also performed with the GE Vivid E9 equipped with an M4S probe. Conventional 2-D and Doppler echocardiography were carried out to exclude any unconfirmed structural disease of the heart including LV hypertrophy, valvular disease, pericardial disease, or cardiomyopathy. EF was obtained by biplane Simpson method [27]. Cine-loop clips of three consecutive cardiac cycles were obtained from the apical 4C, 2C, and 3C view.

### STE imaging analysis

**Fig. 1:**
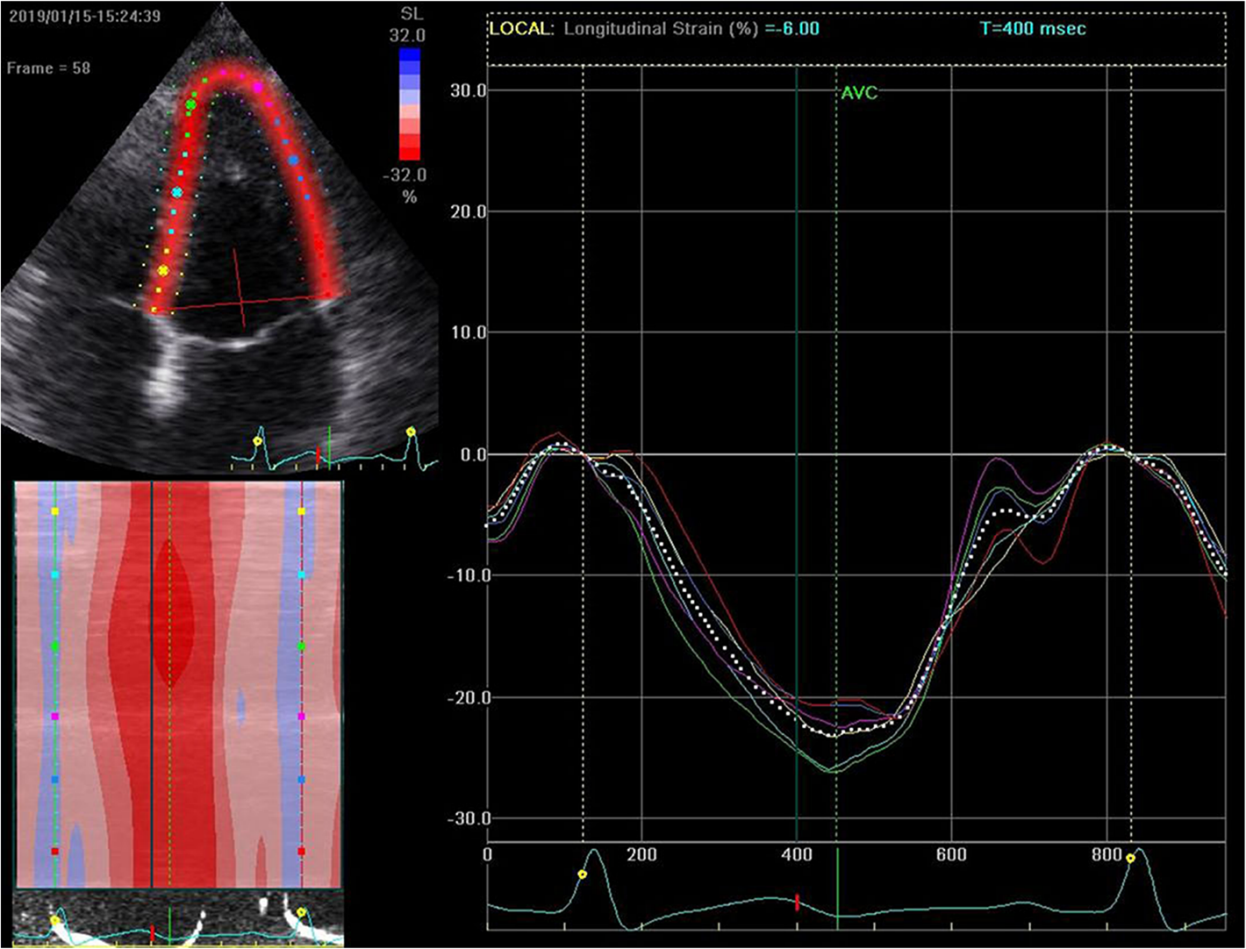
Measurement of GLS of a healthy individual by 2D-STE. The image was obtained from the apical four-chamber view

**Fig. 2:**
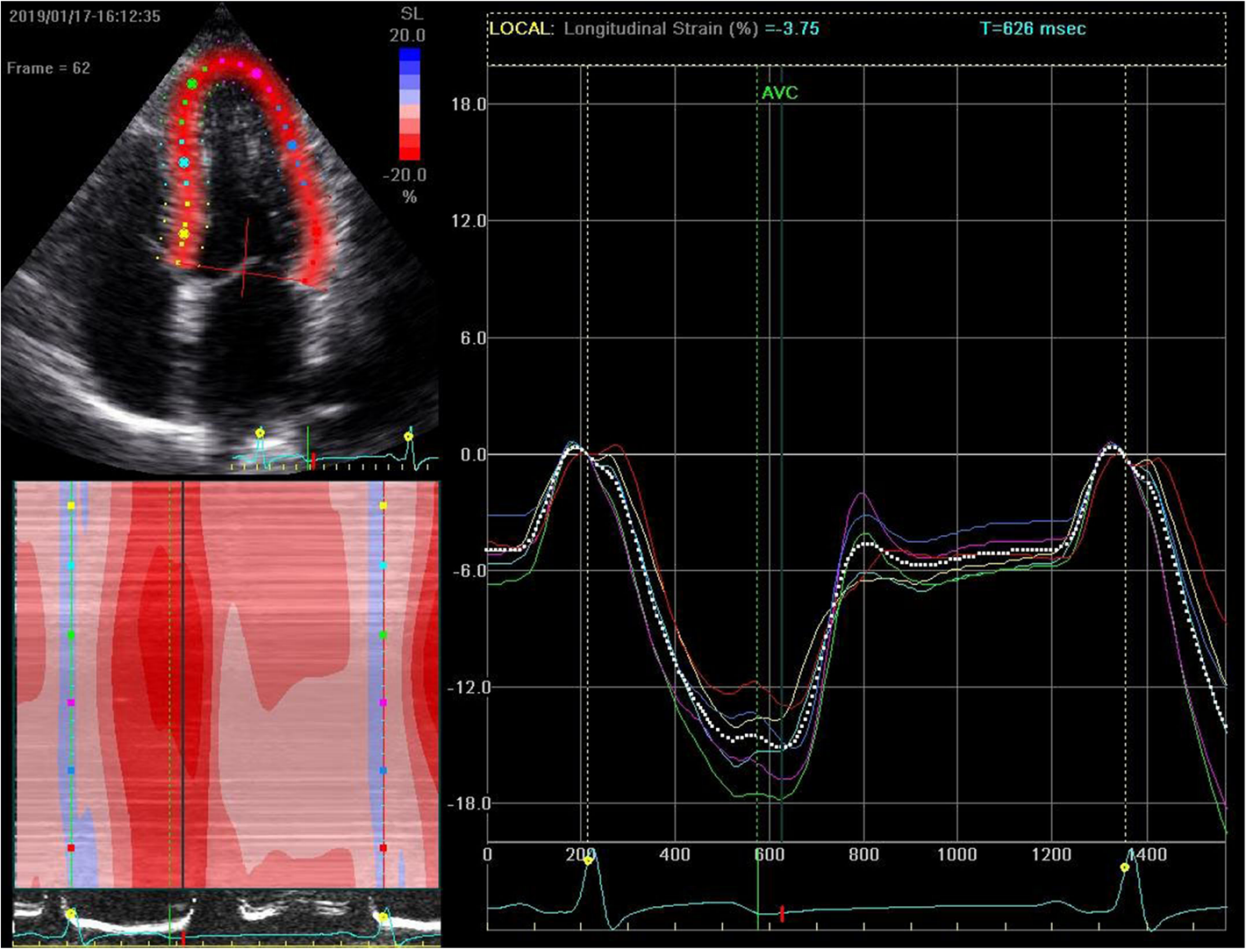
Measurement of GLS of a patient with DN by 2D-STE. The image was obtained from the apical four-chamber view

**Fig. 3:**
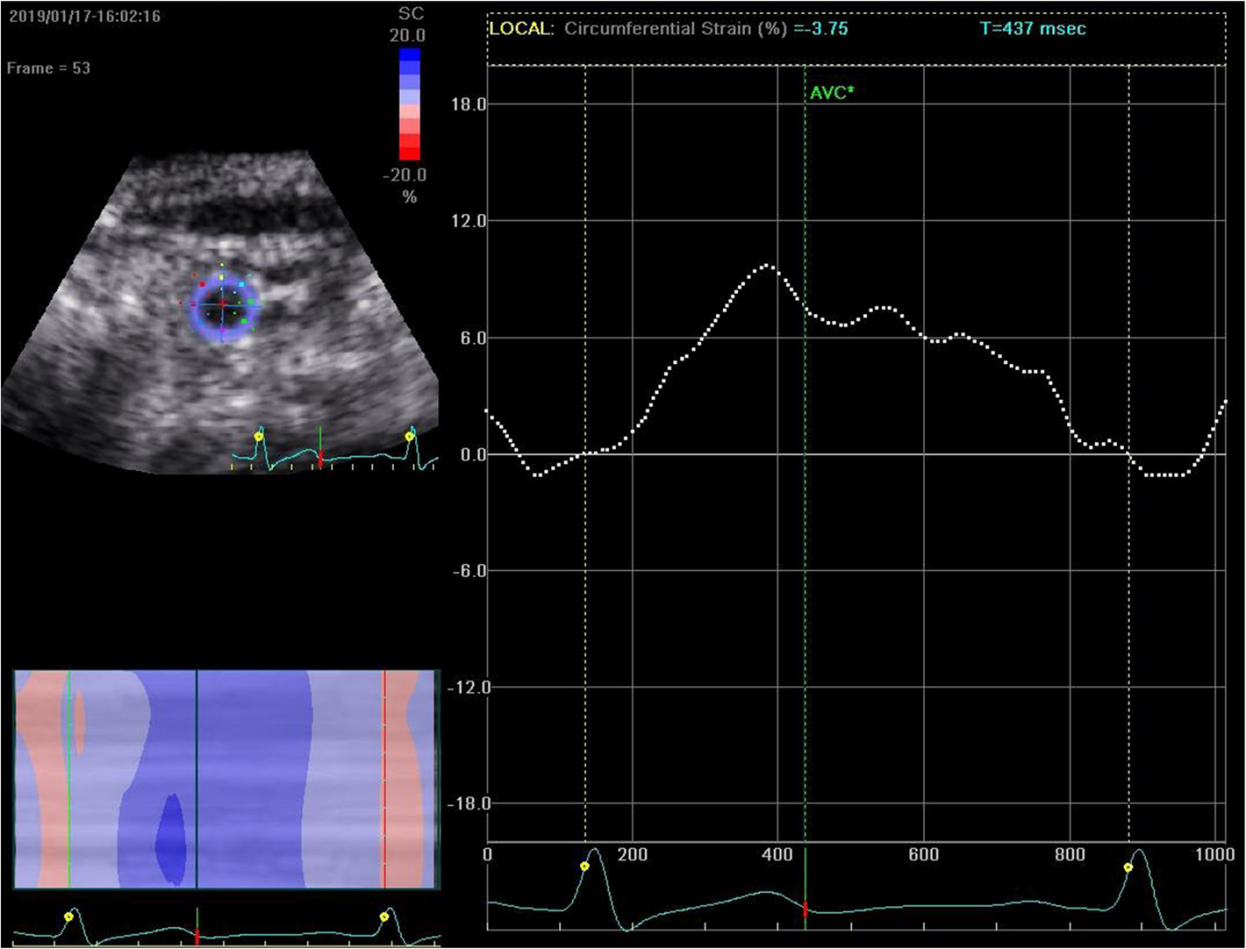
Measurement of CS of a healthy individual by 2D-STE. The image was obtained from the short-axis view of the right common carotid artery

**Fig. 4:**
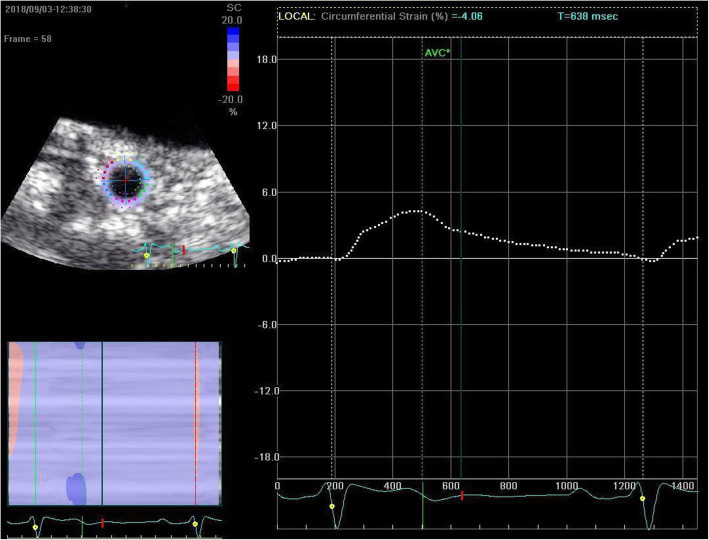
Measurement of CS of a patient with DN by 2D-STE.The image was obtained from the short-axis view of the right common carotid artery

All clips were acquired at 50–70 frames/s. These images were exported from the ultrasound equipment and then analyzed offline with the EchoPAC software (GE Healthcare, IL, USA). The procedure began with manually distinguishing the endocardial at a single frame at end systole, with a region of interest that covers of the myocardial wall thickness (Figs. 1 and 2). The EchoPAC software was used to automatically calculate the strain, including 4CGLS, 2CGLS, and 3CGLS. The circumferential strain (CS) of CCA was also obtained (Figs. 3 and 4).

### Statistical evaluation

Statistical evaluations were carried out using SPSS V 17.0 (SPSS Inc., Chicago, USA). Mean ± SD was used to express quantitative data. Comparisons among the two groups were accomplished with the *t* test of the independent samples. The relationship between CS of the CCA and 4CGLS, 2CGLS, and 3CGLS of the LV was assessed by Pearson’s correlation analysis. *P* < 0.05 was deemed a statistically significant difference.

## Results

### Detailed clinical evaluation versus biochemical test

In the DN group, FBG and glycated HbA1c in the DN group were much higher in contrast to the control group (*p* = 0.000). No significant variation was observed in terms of age, BMI, SBP (systolic blood pressure), DBP (diastolic blood pressure), HR (heart rate), TG, TC, HDL-C, and LDL-C among the two groups (*p* > 0.05) (Table 1).

**Table 1:** Comparison of clinical evaluation and biochemical test

Variables	Group		*p*
	Control (*n* = 112)	DN (*n* = 108)	
AGE (years)	47.41 ± 9.72	50.16 ± 12.30	0.063
BMI (kg/m^2^)	20.82 ± 0.95	21.05 ± 0.95	0.073
SBP (mmHg)	117.11 ± 4.33	118.13 ± 4.89	0.102
DBP (mmHg)	72.13 ± 3.72	72.95 ± 3.93	0.114
HR (bpm)	76.13 ± 5.72	75.04 ± 5.19	0.138
FPG (mmol/l)	5.32 ± 0.41	11.58 ± 2.20	0.000
HbA1c (%)	5.05 ± 0.43	10.38 ± 2.92	0.000
TG (mmol/l)	1.36 ± 0.29	1.31 ± 0.37	0.264
TC (mmol/l)	4.40 ± 0.31	4.44 ± 0.33	0.389
HDL-C (mmol/l)	1.36 ± 0.19	1.32 ± 0.26	0.239
LDL-C (mmol/l)	2.51 ± 0.27	2.48 ± 0.29	0.371

*BMI* body mass index, *SBP* systolic blood pressure, *DBP* diastolic blood pressure, *HR* heart rate, *FBG* fasting blood glucose, *HbA1c* glycated hemoglobin A1c, *TG* triglycerides, *TC* total cholesterol, *HDL-C* high-density lipoprotein cholesterol, *LDL-C* low-density lipoprotein cholesterol

### The carotid ultrasound

There was no significant difference in IMT, PSV, EDV, PI, and RI between the two groups (*p* > 0.05) (Table 2).

**Table 2:** Comparison of conventional ultrasound parameters

Variables	Group		*p*
	Control (*n* = 112)	DN (*n* = 108)	
IMT (mm)	0.73 ± 0.12	0.76 ± 0.13	0.061
PSV (cm/s)	70.48 ± 9.90	67.74 ± 12.26	0.069
EDV (cm/s)	18.67 ± 3.70	18.74 ± 4.08	0.900
PI	1.16 ± 0.11	1.13 ± 0.10	0.058
RI	0.73 ± 0.04	0.72 ± 0.04	0.071
EF (%)	64.41 ± 2.95	63.78 ± 2.35	0.084

*IMT* intima-media thickness, *PSV* peak flow velocity, *EDV* end-diastolic flow velocity, *PI* pulsatility index, *RI* resistance index, *EF* ejection fraction

### STE imaging analysis

The CS in the DN group was lower at the significant levels in contrast to the control group (*p* = 0.000). The 4C GLS, 2C GLS, and 3C GLS in the DN group were lower at significant levels in contrast to the control group (*p* = 0.000, *p* = 0.000, *p* = 0.000) (Table 3).

**Table 3:** Comparison of STE parameters

Variables	Group		*p*
	Control (*n* = 112)	DN (*n* = 108)	
CS (%)	7.71 ± 1.64	4.94 ± 1.45	0.000
4CGLS (%)	− 20.73 ± 1.77	− 17.06 ± 2.92	0.000
2CGLS (%)	− 21.49 ± 1.97	− 16.91 ± 2.98	0.000
3CGLS (%)	− 21.05 ± 2.19	− 16.24 ± 2.55	0.000

*CS* circumferential strain, *GLS* global longitudinal strain, *4C* four-chamber, *2C* two-chamber, *3C* three-chamber

**Fig. 5:**
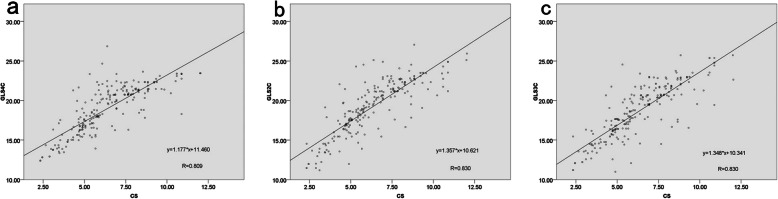
Correlations between the CS and the GLS of apical, four-chamber view (**a**), two-chamber view (**b**), and three-chamber view (**c**)

The correlation coefficient among CS and 4CGLS was 0.809 (*p* = 0.000). The correlation coefficient among CS and 2CGLS was 0.830 (*p* = 0.000). The correlation coefficient among CS and 3CGLS was 0.830 (*p* = 0.000) (Fig. 5).

## Discussion

Atherosclerosis includes structural and functional abnormalities. Carotid IMT has long been regarded as a good indicator of structural abnormalities and can be used to evaluate atherosclerosis [24–26]. Functional abnormalities include a reduction in the distensibility coefficient, an increase in stiffness index, and an incremental increase in elastic modulus [28]. Functional abnormalities occur earlier than structural changes do [28]. 2D-STE has been used to evaluate aortic stiffness [22]. The strain and strain rate obtained from 2D-STE are widely used in the evaluation of myocardial and vascular wall deformation [15, 21, 22]. In this study, the carotid CS in the DN group were lower at significant levels in contrast to the control group, but the difference in IMT among the two groups was not significant. This indicated that the carotid artery stiffness increased in patients with DN, and the functional changes occurred earlier than structural changes, which was consistent with previous studies [28].

The myocardium is divided into three layers: shallow, middle, and deep. The shallow layer is left-handed spiral, the middle layer is circular, and the deep layer is right-handed spiral. The special structure of the myocardial fiber determines that the myocardium has radial contraction, longitudinal contraction, circumferential movement, and rotational movement [29]. Longitudinal contraction of myocardial fibers plays an important role in cardiac movement and has important clinical significance [30]. It was reported that the GLS of the myocardium can be used to evaluate the early systolic function of the heart [30–32]. Radwan and Hussein found a positive correlation at significant levels between GLS and EF, and measurement of GLS using 2D-STE to be accurate and sensitive tool in severe CAD prediction [33]. The study by Shiino et al. showed that GLS is more sensitive than conventional LV EF to detect early improvement in systolic function of the LV after severe aortic valve stenosis patients underwent transcatheter aortic valve implantation and preserved LV systolic function [34]. In our study, the GLS in the DN group were lower at significant levels in contrast to the control group, which indicated that the systolic function of LV was impaired in patients with DN.

Some studies have shown a close relation between arterial stiffness to left ventricular diastolic function [8, 35]. However, there are only few reports on the association between left ventricular systolic function and arterial stiffness. In this study, the outcomes revealed that there was a positive correlation at significant levels between the CS of the carotid artery and the GLS of LV, which indicated that the greater the stiffness of the carotid artery, the greater the impairment of cardiac systolic function. This reason may be that the carotid artery belongs to the elastic artery and is rich in elastic fibers. When LV ejection occurs, the intra-arterial pressure increases, the large artery dilates passively, and the volume increases, so the deformation in the circumferential direction is increased. When arterial stiffness increases, the mean elasticity decreases, the burden of the LV contraction is increased, and the systolic function is impaired.

In conclusion, increased arterial stiffness measured by 2D-STE is indicative of a potential link between vascular changes and LV systolic function. When the CS decreases, the GLS is impaired. The CS has a certain predictive effect on the early reduction of LV systolic function. 2D-STE can detect the relationship between CS and GLS in the early stage.

## Data Availability

The analyzed and/or used datasets in this study can be obtained on reasonable request to the corresponding author.

## Abbreviations

2C: Two-chamber
2D-STE: Two-dimensional speckle-tracking strain echocardiography
3C: Three-chamber
4C: Four-chamber
BMI: Body mass index
CAD: Coronary artery disease
CCA: Common carotid artery
CS: Circumferential strain
CVD: Cardiovascular disease
DBP: Diastolic blood pressure
DM: Diabetes mellitus
DN: Diabetic nephropathy
EDV: End-diastolic flow velocity
FBG: Fasting blood glucose
GLS: Global longitudinal strain
HbA1c: Glycated hemoglobin A1c
HDL-C: High-density lipoprotein cholesterol
HR: Heart rate
IMT: Intima-media thickness
LDL-C: Low-density lipoprotein cholesterol
LV: Left ventricle
PI: Pulsatility index
PSV: Peak flow velocity
RI: Resistance index
SBP: Systolic blood pressure
TC: Total cholesterol
TG: Triglycerides
